# Down regulation of human positive coactivator 4 suppress tumorigenesis and lung metastasis of osteosarcoma

**DOI:** 10.18632/oncotarget.18290

**Published:** 2017-05-30

**Authors:** Xu Hu, Chao Zhang, Ying Zhang, Christopher S. Hong, Wugui Chen, Weiwei Shen, Hongkai Wang, Jianrong He, Pei Chen, Yue Zhou, Chunmeng Shi, Tongwei Chu

**Affiliations:** ^1^ Department of Orthopedics, Xinqiao Hospital, Third Military Medical University, Chongqing, 400037, China; ^2^ Institute of Combined Injury, State Key Laboratory of Trauma, Burns and Combined Injury, Chongqing Engineering Research Center for Nanomedicine, College of Preventive Medicine, Third Military Medical University, Chongqing, 400038, China; ^3^ The Ohio State University, College of Medicine, Columbus, OH, 43210, USA

**Keywords:** PC4, SP1, MMP, pulmonary metastasis

## Abstract

Osteosarcoma is a kind of primary malignant bone tumor with the highest incidence and an extraordinarily poor prognosis and early pulmonary metastasis formation as a frequent occurrence. Transcriptional positive coactivator 4 (PC4) has multiple functions in DNA replication, transcription, repair and chromatin organization, even in tumorigenesis. However, the precise function of PC4 in osteosarcoma is still unclear and controversial. In this paper we found PC4 was upregulated in patient-derived osteosarcoma tissues compared to normal. Moreover, higher expression of PC4 was correlated with poorer overall survival and advanced clinicopathological tumor staging. Down regulation of PC4 in the highly metastatic osteosarcoma cells reduced the malignant behaviors *in vitro* and *in vivo*. Analyzing the downstream genes affected obviously by shPC4 with RNA sequencing, we found knocking down PC4 will inhibit the propensity for lung metastasis through transcriptional suppression of MMPs pathways. Taken together, PC4 may be an attractive therapeutic strategy for osteosarcoma, especially in preventing lung metastasis formation.

## INTRODUCTION

Approximately 20% of osteosarcoma patients exhibit lung metastasis when diagnosed, while an additional 40% develop lung metastasis in advanced stages, which correlates with poor 5-year survival rates [1]. Despite intense efforts to characterize the genomic patterns of osteosarcoma, effective therapeutic targets and diagnostic markers are still in urgent need [2]. The purpose of this paper is to find a possible therapeutic target for osteosarcoma.

Positive coactivator 4 (PC4) is a highly conserved DNA-binding nuclear protein and is involved in distinct DNA-dependent processes, such as DNA repair, replication, and transcription [3–6]. Previously, we reported that PC4 was a novel oncogenic gene whose overexpression accompanied with the malignant transformation of dermis-derived mesenchymal stem cells [7, 8]. PC4 was found to highly expressed in human prostate stromal cells at the embryonic stage and declined to significantly lower levels by adulthood, while elevated in prostate cancer associated stroma [7]. As osteosarcoma is believed to originate from mesenchymal cells, we sought to evaluate the potential function of PC4 in osteosarcoma tumorigenesis and pulmonary metastasis. In this context, it has been demonstrated that high PC4 expression in osteosarcoma correlates with poor prognosis, and suppression of PC4 blocked the pulmonary metastasis by reducing malignancy phenotype through transcriptional level depression of MMP9. Our findings imply that PC4 may be an effective potential therapeutic gene to inhibit osteosarcoma tumorigenesis and prevent lung metastasis.

## RESULTS

### High PC4 expression in osteosarcoma correlates with poor patient prognosis

**Figure 1:**
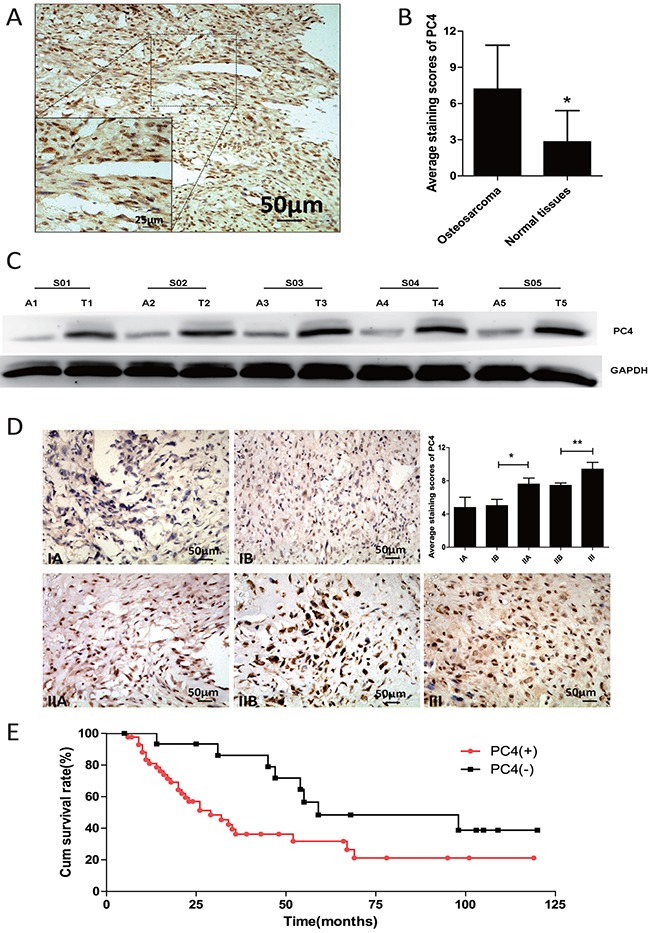
Increased expression of PC4 protein in osteosarcoma tissues **(A)** Immunohistochemical staining of PC4 expression in osteosarcoma. PC4 was mainly located in cell nucleus. **(B)** Average immnohistochemical staining scores of PC4 expression from 198 osteosarcoma tissues and 44 adjacent normal tissues. PC4 was highly expressed in osteosarcoma. (*p < 0.01). **(C)** Western blotting analysis of PC4 expression of 5 osteosarcoma patients (A1-A5) and the adjacent normal tissues (T1-T5). **(D)** Immnohistochemical staining analysis of PC4 expression in different pathological stages. IIA group was higher than IB group (*p < 0.05), III group was higher than IIA group (**p < 0.05). **(E)** Statistical analysis of correlation between PC4 expression level and the survival of osteosarcoma patients (PC4^+^, n=43; PC4^−^, n=16; *p < 0.01)* (Kaplan-Meier survival curves).

We analyzed 5 osteosarcoma tissues and adjacent normal counterparts by western-blot, PC4 was differentially overexpressed in osteosarcoma (Figure 1C). To confirm this, immunohistochemistry staining was performed in additional patient-derived paraffin-embedded osteosarcoma samples (n=82) and tissue microarray sections (n=116) and compared to normal bone tissues of healthy controls (n=44). The average staining score in osteosarcoma was significantly higher than normal tissues (7.2±0.26 vs 2.8±0.39, p<0.01, Figure 1B), and PC4 staining localized primarily to the nucleus (Figure 1A). The data gathered in this part implied that PC4 was increased in osteosarcoma.

Then we compared immunohistochemical staining across tumors of different clinical staging. Greater PC4 expression in advanced stage tumors was found (Figure 1D). High PC4 expression was detected in 160 of 198 cases and it was correlated with staging and tumor size (Table 1). Interestingly, the prevalence of PC4 positivity was higher in patients with pulmonary metastasis (Enneking stage III, 85%) compared to total (80%), suggesting PC4 might be involved in osteosarcoma metastatic potential.

**Table 1:** Relationship between PC4 expression and clinicopathological characteristics of osteosarcoma patients

	PC4		*p*
	Low or none	High	
Total osteosarcoma patients	38	160	
Sex			>0.05
Male	15	71	
Female	23	89	
Age			>0.05
< 40	25	125	
≥ 40	13	35	
Enneking stages			<0.01
IA	4	5	
IB	11	15	
IIA	5	22	
IIB	15	101	
III	3	17	
Tumor size			<0.01
≤ 8 cm	21	51	
> 8 cm	17	109	

Follow-up data were available from 59 patients. Statistically difference in survival was found between the PC4 positive group (n=43) and the PC4 low or negative group (n=16) (p<0.05) (Figure 1F).

### Association of PC4 with clonogenicity and tumorigenicity of osteosarcoma cell lines

**Figure 2:**
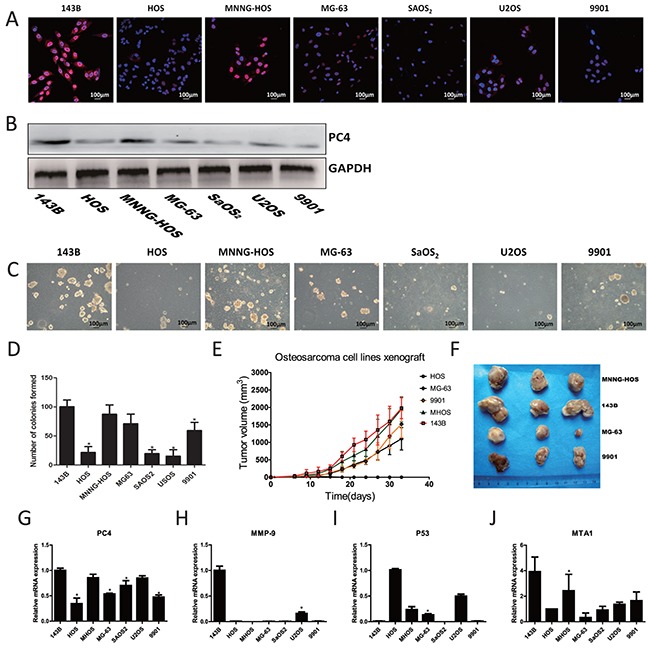
Expression of PC4 in osteosarcoma cells and malignant phenotype of different osteosarcoma cell lines **(A)** Immunofluorescent staining of PC4. PC4 (red), DAPI (blue). **(B)** Western blotting analysis of PC4 expression level in seven osteosarcoma cell lines. GAPDH served as control. **(C, D)** Spheroid development in semisolid soft agar medium after 7 days, which cells were grown in six-well plates with triple replications. **(E, F)** Tumorigenicity of five osteosarcoma cell lines. Grow for 5 weeks in nude mice (n=3 mice in each group). Mice were injected with 5*10^6^ cells. All the cells developed a visible tumor except HOS group. **(G-J)** Quantitative RT-PCR test of PC4 and malignant phenotype related genes in different osteosarcoma cell lines (*comparing with 143B *p<0.05*).

To characterize the functional relevance of increased PC4 expression in osteosarcoma, we performed further experiments *in vitro* in seven osteosarcoma cell lines. Immunofluorescent staining for PC4 demonstrated prominent nuclear localization and exhibited greatest intensity in 143B and MNNG-HOS cells (Figure 2A). Western-blotting and real-time PCR confirmed these findings, demonstrating highest PC4 expression in 143B and MNNG-HOS cells, moderate PC4 levels in MG63 and U2OS cells, and lowest PC4 expression in HOS, SAOS2, and 9901 cells (Figure 2B). Clonogenicity assays demonstrated greatest sphere formation in 143B cells, followed by moderate formation in MNNG-HOS, MG63, and 9901 cells, and lowest formation in HOS, U2OS, and SAOS2 cells (Figure 2C). Likewise, 143B and MNNG-HOS cells exhibited greatest tumorigenicity in xenografted mouse models, while MG63 and 9901 cells had lower tumorigenicity; HOS xenografts did not develop visible tumors in our experiment (Figure 2D). Based on these results, 143B cells were selected for further research, based on the elevated PC4 levels and high potential for lung metastases [9, 10]. These results indicated that PC4 expression was possibly related to clonogenicity *in vitro* and tumorigenicity *in vivo*. We also found that 143B cells had the highest expression of MMP-9, which may have been related to the propensity for lung metastases, observed in 143B cells. Compared to other osteosarcoma cell lines, mRNA levels of P53 were extraordinary low in 143B cells (Figure 2G-2J).

### Knockdown of PC4 further decreases the malignancy of 143B cells

**Figure 3:**
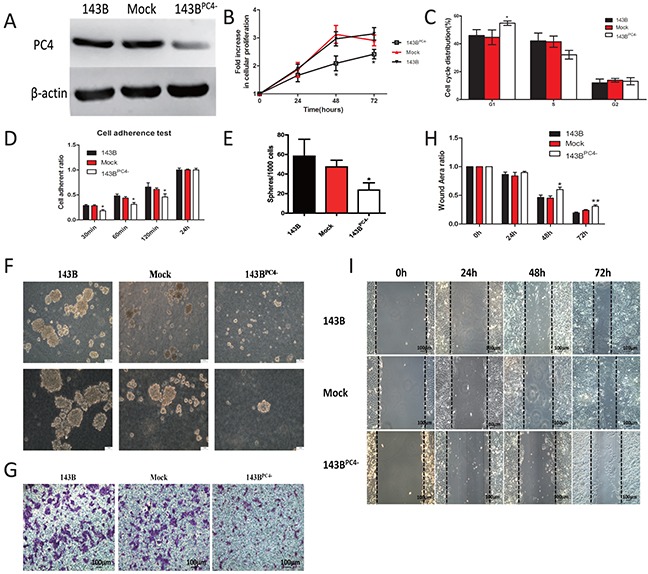
Stable knockdown of PC4 and the accompanied malignant phenotype change **(A)** Western blotting analysis of PC4 knockdown efficiency in 143B cells. **(B)** Cell proliferation assays show slight inhibition by lv-shRNA-PC4 (*p<0.01). **(C)** Cell cycle distribution of 143B cells with lv-shRNA transfection (*p<0.05). **(D)** Cell attachment assay. 2*10^4^ cells were seeded into 96-well culture plates, and incubated at 37°C for 30 min or 60 min or 120min or 24 hours. After incubation, unattached cells were washed with PBS, and adherent cells were counted with CCK-8 kits, (*p<0.01). **(E, F)** Spheroid development in semisolid soft agar medium after 7 days. 143B showed decreased efficiency of sphere-forming in 143B^PC4−^group. Spheres were counted in five random fields of vision. **(G)** Invasion assay. 143B cells seeded on the upper chamber with pre-coated matrigel for 24 hours. Cells on the underside of the membrane were fixed, stained with crystal violet. **(H, I)** Effect of lv-shRNA-PC4 on 143B cell migration by wound-healing assay. The 143B cells were seeded in 6-well plates for 24h, after cell reached 100% density wounds were created. Cell migration was observed at 0h, 24h, 48h, and 72h after wounding. The migration distance was calculated as the width at indicated time (*p<0.05 versus control).

To further investigate the role of PC4 in the malignancy of 143B cells, PC4 was silenced in 143B utilizing lentivirus shRNA. Stably transfected 143B^PC4−^ cells were obtained, which PC4 expression level was maintained at 30% comparing with the parental cells (Figure 3A).

Cell proliferation was decreased after PC4 knockdown (Figure 3B). In 143B^PC4−^ group, the percentage of cells in G1 phase increased (p<0.05) while those in S phase decreased but there was no significant difference, the data suggested down regulation of PC4 in 143B cells induces G1-phase arrest (Figure 3C). The 143B^PC4−^ group showed slower adherent speeds at 30min, 60min and 120min (Figure 3D). Likewise, 143B^PC4−^ cells showed decreased colony formation ability (Figure 3E, 3F). Transwells suggested that shPC4 may reduce the invasion of 143B (Figure 3G). Scarification test results demonstrated that migration potential was inhibited in 143B^PC4−^ cells (Figure 3H, 3I).

### Down regulation of PC4 in 143B cells suppresses tumor growth *in vivo* and development of pulmonary metastases

**Figure 4:**
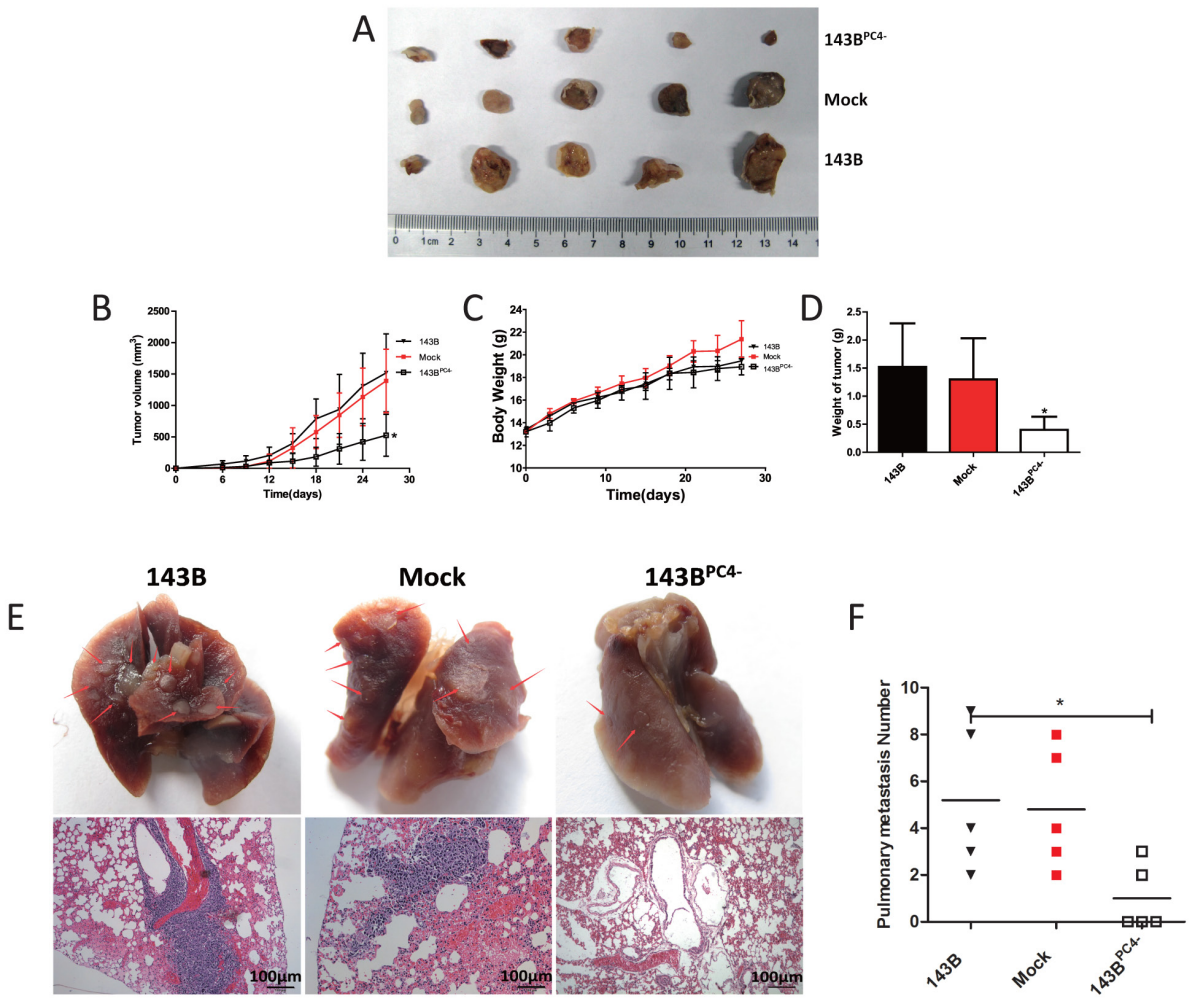
Tumorigenicity of osteosarcoma cells with stable knockdown of PC4 **(A)** Excised all tumors in nude mice at day 27 when the biggest tumor volume reach 2000 mm^3^. **(B)** Tumor volume of 143B xenografts. 143B^PC4−^ group had a regression compared with control (*P<0.05). **(C)** Body weight of nude mice. **(D)** Excised Tumor weight (*P<0.05). **(E)** Excised lung in nude mice when tumor volume reach 2000 mm^3^. Photographs of lungs of each group. Arrow shows the metastasis. Representative lungs H&E staining of each group. **(F)** Visible lung metastasis number of each group (*P<0.05).

Mean tumor sizes in the parental 143B group, mock-transfected 143B group, and 143B^PC4−^ group were 1 519±620 mm^3^, 1 390±504 mm^3^, and 525±333 mm^3^, respectively (means±SD, P<0.05; Figure 4A, 4B). The respective weights of the tumors were 1.52±0.77 g, 1.30±0.73 g, and 0.40±0.23 g (means±SD, P<0.05; Figure 4D). These data suggested that the growth might be suppressed in 143B^PC4−^ *in vivo* without affecting body weight.

To determine the role of PC4 in pulmonary metastasis in osteosarcoma, and to avoid the effect on proliferation, another 15 nude mice were used. Lungs were excised when tumors reached 2 000mm^3^. Both the rate of pulmonary metastasis and the number of visible metastases in the 143B^PC4−^ group were dramatically reduced (Figure 4E, 4F). Diagnoses of metastatic nodules were confirmed by H&E stains.

### RNA-seq analysis reveals diminished MMP expression after PC4 knockdown

We utilized RNA-seq to explore the molecular alterations after PC4 interference in 143B cells. In total, 12562108 reads were obtained in 143B cells and 129963468 reads were obtained in 143B^PC4−^ cells. We mapped 87.63% of the reads to the human reference genome (hg18) in 143B cells and 88.06% in 143B^PC4−^cells. In comparison with 143B cells, 572 genes were increased and 513 genes were decreased in 143B^PC4−^ cells. Top 10 up and down represented genes were shown (Table 2). CXCL1, MMP9, IL1B, WNT7A, and CCL2 were associated with cancer malignant characteristics which were remarkably decreased in Top 10 list. MMP9 (log_2_Ratio=-8.19), MMP2 (log_2_Ratio=-1.02) and FN (log_2_Ratio=-3.99) were downregulated in 143B^PC4−^ cells, which were strongly associated with metastasis and had reciprocal actions. KEGG pathway of enrichment analysis of differentially expressed genes was performed, and the top 20 pathway enrichments for 143B^PC4−^ cells were shown, comparing with all genes with pathway annotation, pathways which P<0.05 were listed (Table 3). Gene ontology functional classification of differentially expressed genes was analyzed, cluster frequency comparing with genome frequency, corrected P<0.05 were listed (Table 4).

**Table 2:** Top 10 up- and down- represented genes after PC4 knocking down in 143B cell

Gene	GeneID	Description	log2 Ratio	Gene_length	P-value	FDR
DRD5	1816	Dopamine receptor D5	7.80382017	2398	2.67E-13	2.84E-12
INHBE	83729	Inhibin, beta E	6.757445414	2453	1.22E-61	6.70E-60
CTH	1491	Cystathionase (cystathionine gamma-lyase)	6.182881406	2140	0	0
FAM90A1	55138	Family with sequence similarity 90, member A1	6.07740762	2516	0.000102221	0.000435026
MEIS1-AS3	730198	An RNA Gene, and is affiliated with the non-coding RNA class.	5.915134907	4115	1.48E-06	8.22E-06
LGR6	59352	Leucine-rich repeat containing G protein-coupled receptor 6	5.825052551	3458	2.49E-05	0.000117228
MKX	283078	Mohawk homeobox	5.736785943	3658	3.16E-15	3.88E-14
ESRP1	54845	Epithelial splicing regulatory protein 1	5.480263981	3806	0.000102221	0.000434922
ATF3	467	Activating transcription factor 3	4.908885316	2400	6.00E-127	7.54E-125
CHAC1	79094	ChaC, cation transport regulator homolog 1 (E. coli)	4.469583903	1578	1.31E-248	4.56E-246
CXCL1	2919	Chemokine (C-X-C motif) ligand 1	−10.12824838	1207	4.70E-32	1.28E-30
KRT75	9119	Keratin 75	−8.365650472	2125	5.59E-17	7.69E-16
SERPINA3	12	Serpin peptidase inhibitor, clade A	−8.215694509	1629	5.93E-12	5.67E-11
MMP9	4318	Matrix metallopeptidase 9	−8.197914746	2387	5.59E-17	7.69E-16
IL1B	3553	Interleukin 1, beta	−8.158410629	1498	2.60E-169	5.12E-167
WNT7A	7476	Wingless-type MMTV integration site family, member 7A	−7.923708789	1732	1.78E-10	1.51E-09
STRA6	64220	Stimulated by retinoic acid gene 6 homolog (mouse)	−7.923525266	3097	3.68E-18	5.42E-17
CCL2	6347	Chemokine (C-C motif) ligand 2	−7.664093493	760	3.47E-60	1.84E-58
BCHE	590	Butyrylcholinesterase	−7.620436455	2461	5.93E-12	5.66E-11
NAP1L3	4675	Nucleosome assembly protein 1-like 3	−7.454490225	2761	5.93E-12	5.66E-11

**Table 3:** KEGG pathway enrichment analysis of different express genes

Pathway	DEGs withpathwayannotation(906)	All geneswith pathwayannotation(17252)	P-value	Q-value	Pathway IDof KEGG
p53 signaling pathway	23 (2.54%)	143 (0.83%)	1.52172E-06	0.000413907	ko04115
Axon guidance	34 (3.75%)	308 (1.79%)	3.53746E-05	0.004810942	ko04360
Isoquinoline alkaloid biosynthesis	6 (0.66%)	16 (0.09%)	0.000104847	0.009506101	ko00950
Glycine, serine and threonine metabolism	12 (1.32%)	71 (0.41%)	0.0002982	0.019414805	ko00260
Tropane, piperidine and pyridine alkaloid Biosynthesis	5 (0.55%)	13 (0.08%)	0.00035689	0.019414805	ko00960
Malaria	12 (1.32%)	76 (0.44%)	0.000568131	0.025755258	ko05144
Fatty acid biosynthesis	5 (0.55%)	15 (0.09%)	0.000762581	0.029631699	ko00061
Tyrosine metabolism	12 (1.32%)	81 (0.47%)	0.001018911	0.034642974	ko00350
Phenylalanine metabolism	7 (0.77%)	33 (0.19%)	0.001383711	0.041818821	ko00360
MAPK signaling pathway	36 (3.97%)	425 (2.46%)	0.003285155	0.083304921	ko04010
Legionellosis	13 (1.43%)	105 (0.61%)	0.003368949	0.083304921	ko05134
Alanine, aspartate and glutamate metabolism	8 (0.88%)	57 (0.33%)	0.009438303	0.213934868	ko00250
Neurotrophin signaling pathway	22 (2.43%)	248 (1.44%)	0.01151266	0.240880271	ko04722
Cytokine-cytokine receptor interaction	26 (2.87%)	317 (1.84%)	0.01654431	0.300741515	ko04060
Methane metabolism	7 (0.77%)	51 (0.3%)	0.01658501	0.300741515	ko00680
NOD-like receptor signaling pathway	14 (1.55%)	144 (0.83%)	0.01932437	0.315335493	ko04621
Measles	17 (1.88%)	188 (1.09%)	0.02056757	0.315335493	ko05162
Cocaine addiction	10 (1.1%)	91 (0.53%)	0.02086779	0.315335493	ko05030
Vitamin B6 metabolism	3 (0.33%)	12 (0.07%)	0.02223889	0.318367267	ko00750
Carbon fixation in photosynthetic organisms	5 (0.55%)	32 (0.19%)	0.02445567	0.332597112	ko00710
ECM-receptor interaction	22 (2.43%)	269 (1.56%)	0.02667029	0.345443756	ko04512
Rheumatoid arthritis	11 (1.21%)	115 (0.67%)	0.0392752	0.485584291	ko05323

**Table 4:** Gene ontology functional classification of differentially expressed genes

Gene Ontology term	Cluster frequency	Genome frequency of use	Corrected P-value
Molecular function			
Protein binding	299 out of 837 genes, 35.7%	4282 out of 15165 genes, 28.2%	0.00026
Binding	710 out of 837 genes, 84.8%	12079 out of 15165 genes, 79.7%	0.0168
Cellular component			
Cell junction	37 out of 858 genes, 4.3%	351 out of 16090 genes, 2.2%	0.01023
Extracellular matrix	33 out of 858 genes, 3.8%	312 out of 16090 genes, 1.9%	0.0488
Biological process			
Locomotion	91 out of 811 genes, 11.2%	838 out of 14596 genes, 5.7%	6.13E-07
Anatomical structure development	232 out of 811 genes, 28.6%	2997 out of 14596 genes, 20.5%	1.73E-05
Response to chemical stimulus	163 out of 811 genes, 20.1%	1934 out of 14596 genes, 13.3%	2.28E-05
Developmental process	262 out of 811 genes, 32.3%	3517 out of 14596 genes, 24.1%	4.81E-05
Cell motility	62 out of 811 genes, 7.6%	538 out of 14596 genes, 3.7%	5.36E-05
Localization of cell	62 out of 811 genes, 7.6%	538 out of 14596 genes, 3.7%	5.36E-05
Response to external stimulus	81 out of 811 genes, 10.0%	787 out of 14596 genes, 5.4%	6.90E-05
Cellular component movement	62 out of 811 genes, 7.6%	568 out of 14596 genes, 3.9%	0.00038
Response to lipid	47 out of 811 genes, 5.8%	382 out of 14596 genes, 2.6%	0.00038
Multicellular organismal development	204 out of 811 genes, 25.2%	2681 out of 14596 genes, 18.4%	0.00075
Positive regulation of biological process	135 out of 811 genes, 16.6%	1642 out of 14596 genes, 11.2%	0.00215
Cell migration	49 out of 811 genes, 6.0%	431 out of 14596 genes, 3.0%	0.00226
System development	184 out of 811 genes, 22.7%	2407 out of 14596 genes, 16.5%	0.00252
Signaling	281 out of 811 genes, 34.6%	3999 out of 14596 genes, 27.4%	0.003
Response to stimulus	351 out of 811 genes, 43.3%	5195 out of 14596 genes, 35.6%	0.00318
Single-organism developmental process	153 out of 811 genes, 18.9%	1940 out of 14596 genes, 13.3%	0.00433
Anatomical structure morphogenesis	106 out of 811 genes, 13.1%	1236 out of 14596 genes, 8.5%	0.00554
Response to oxygen levels	9 out of 811 genes, 1.1%	25 out of 14596 genes, 0.2%	0.00701
Response to organic substance	99 out of 811 genes, 12.2%	1141 out of 14596 genes, 7.8%	0.00736
Positive regulation of cellular process	114 out of 811 genes, 14.1%	1364 out of 14596 genes, 9.3%	0.00772
Cell proliferation	77 out of 811 genes, 9.5%	836 out of 14596 genes, 5.7%	0.01147
Cellular developmental process	135 out of 811 genes, 16.6%	1706 out of 14596 genes, 11.7%	0.01616
Anatomical structure formation involved in morphogenesis	48 out of 811 genes, 5.9%	450 out of 14596 genes, 3.1%	0.01683
Biological regulation	421 out of 811 genes, 51.9%	6523 out of 14596 genes, 44.7%	0.0206
Intracellular signal transduction	96 out of 811 genes, 11.8%	1125 out of 14596 genes, 7.7%	0.02067
Regulation of metabolic process	198 out of 811 genes, 24.4%	2717 out of 14596 genes, 18.6%	0.0225
Enzyme linked receptor protein signaling pathway	66 out of 811 genes, 8.1%	698 out of 14596 genes, 4.8%	0.02319
Response to organic cyclic compound	44 out of 811 genes, 5.4%	405 out of 14596 genes, 2.8%	0.02487
Response to molecule of bacterial origin	23 out of 811 genes, 2.8%	157 out of 14596 genes, 1.1%	0.02983
Chemotaxis	44 out of 811 genes, 5.4%	408 out of 14596 genes, 2.8%	0.02987

### Down regulation of PC4 inhibits the transcription of MMP9 through the synergy with SP1

**Figure 5:**
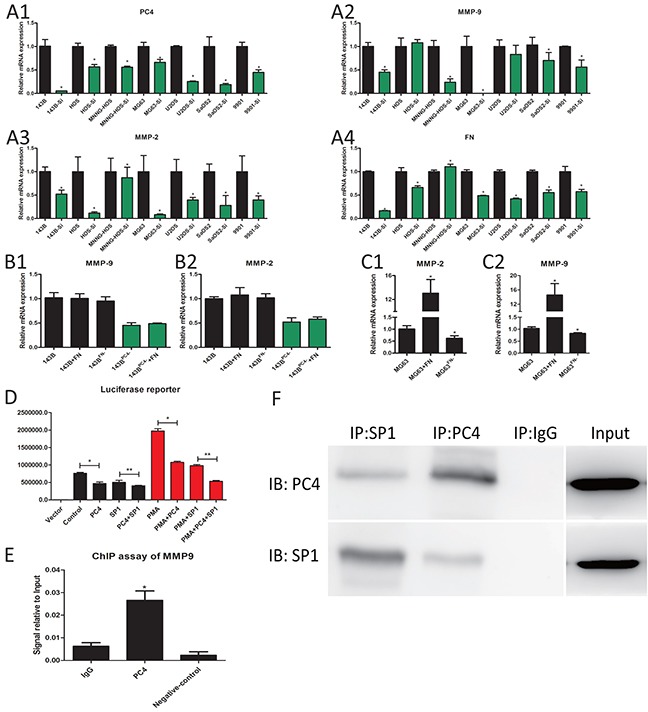
PC4 binds with SP1 and regulate the transcription of MMP9 **(A)** The mRNA expression levels of PC4, MMP9, MMP2, and FN after lv-shRNA-PC4 transfected in seven osteosarcoma cells. All the cell lines were compared to parental cells respectively (*P<0.05). **(B)** 143B treated with 35ug/ml FN protein for 4h or FN siRNA, the mRNA expression levels of MMP9, MMP2 (*P<0.05). **(C)** MG63 treated with FN protein or FN siRNA, the mRNA expression levels of MMP9, MMP2 (*P<0.05). **(D)** Luciferase reporter. PMA was used as an inducer for MMP9 luciferase activity. Both PC4 siRNA and SP1 siRNA decrease the luciferase activity of MMP9 promoter region, and PC4 siRNA and SP1 siRNA have the combined effect (*P<0.05, **P<0.05). **(E)** Quantitative ChIP assay of endogenous PC4 interact with MMP9 promoter region in 143B cells. MMP9 cDNA was detectable in the immunoprecipitated chromatin samples of 143B cells using a PC4 antibody, suggesting PC4 binds to the MMP9 promoter region. The same amount of isotype antibody was used as control (IgG), as well as no antibody controls (Negative control). RT-PCR results are expressed as percentages of the total input DNA (*P<0.05). **(F)** PC4 binds to SP1 in 143B. 143B cell extracts protein was used. Co-Immunoprecipitation was performed using PC4 antibody and SP1 antibody, IgG as control.

In various osteosarcoma cells, PC4 knockdown reduced MMP9 and MMP2 mRNA levels, compared to each parental cell respectively (Figure 5A1-5A4). RNA-seq data showed fibronectin and MMP9 and MMP2 were reduced in 143B^PC4−^ cells. As both exogenously added fibronectin and endogenous up-regulation of fibronectin can result in an increase in MMPs according to the references [11–13], we infer PC4 might affect the MMPs through the FN. In order to delineate the mechanisms underlying downregulation of MMP9 and MMP2 after PC4 knockdown, and to confirm that these results were not restricted to the 143B tumor cell line, we measured fibronectin mRNA levels in seven osteosarcoma cell lines after PC4 knockdown. The mRNA levels of fibronectin were reduced in all tested cell lines except MNNG-HOS (Figure 5A4). Then exogenous human plasma fibronectin purified protein or FN siRNA were added to 143B, MG63 and 143B^PC4−^ cells to test the effect of fibronectin on MMP9 and MMP2, however, the mRNA levels of both MMP2 and MMP9 showed no obvious changes in either 143B or 143B^PC4−^ cells (Figure 5B1, 5B2) but dramatical changes in MG63 (Figure 5C1, 5C2). These results showed that the MMP9 and MMP2 in 143B cells were not sensitive to the fibronectin in mRNA level. As such, there may be another mechanism for the regulation of MMP2 and MMP9 in 143B cells after PC4 knockdown. All in all, our results support previous work demonstrating that MMP9 is strongly implicated in lung metastasis potential and invasive behavior in osteosarcoma [14–18] and this may occur in a PC4-dependent manner.

PC4 usually functioned as a co-activator in transcription, and FN had no obvious stimulative effect on MMP9 in 143B, so we hypothesized that PC4 directly increases the MMP9 expression at the transcriptional level. As such, SP1 is the well known transcription factor of MMP9 [19, 20] and we tested whether there was synergy between SP1 and PC4 in MMP9 regulation. SP1 siRNA and PC4 siRNA can both reduce the luciferase activity of MMP9 promoter, and this effect was more obvious under the PMA stimulation [21]. These results implied that SP1 and PC4 were involved in the transcription promotion of MMP9 (Figure 5D). ChIP analysis suggested that PC4 protein bound to the promoter region of MMP9 (Figure 5E). To confirm the combination between PC4 and SP1, we utilized Co-immunoprecipitation assays. SP1 could be detected in the samples immunoprecipitated using PC4 antibody, PC4 could be detected in the samples immunoprecipitated using SP1 antibody (Figure 5F). In conclusion, PC4 plays a synergistic role by forming SP1 transcription complex and then activate the transcription of MMP9.

## DISCUSSION

Although great progress has been made for the treatment in osteosarcoma in the recent years, new effective biomarkers and gene therapeutic targets are still urgently needed. We have previously identified PC4 was increased in adult mesenchymal stem cells after spontaneous transformation [8]. Some researches imply osteosarcoma may originate from malignant transformed mesenchymal stem cells [22, 23]. Our preliminary experiment showed PC4 was increased in osteosarcoma and more intense staining of PC4 was observed in specimens with metastasis. Taking all the clues together, PC4 seems to be the cause of cancer malignant transformation but the evidence is insufficient and the mechanism is unclear.

PC4 is a non-histone chromatin-associated protein, and generally described as a co-activator for RNA polymerase (Pol) II and Pol III transcription *in vitro* and might has a dual role in gene expression. PC4 has a conserved unique non-specific DNA binding domain and affects DNA repairing, replication, and transcription [5, 24]. The co-activator domain (22-91aa) of PC4 and DNA binding domain of SP1 are critical for PC4/SP1 interaction, and PC4 phosphorylation negatively suppressed this interaction [25]. PC4 Plays dual role in transcription initiation [3], knocking down PC4 in HeLa cells opens up the chromatin and upregulate several genes suggesting that there might be a repressive role for PC4 in vivo [26], and this is echoed by our RNA-seq data with 572 genes upregulated after PC4 suppression. PC4 stimulates reconstituted basal transcription at low PC4 levels and represses transcription when PC4 increased [27]. PC4 has been recently implicated in several cancers [28–30], acting as a tumor promoter, including pancreatic cancer, small and non-small cell lung cancers [31], and astrocytoma [32].

As early lung metastasis of osteosarcoma is critical in prognosis and treatment, we mainly focused on the counter-effects upon lung metastasis after PC4 suppression. Our clinical analysis revealed a correlation between PC4 expression with poor prognosis and metastases and advanced tumor staging in osteosarcoma. Likewise, PC4 was upregulated in osteosarcoma tissues compared to the adjacent normal tissues. These results of PC4 are coincident with researches in other tumors, which PC4 may be a tumor promoter. The PC4 expression in various osteosarcoma cell lines implies the relevance between PC4 and the malignant characteristics. 143B exhibited the greatest malignant characteristics and the highest expression of PC4. As 143B and MNNG-HOS were derived from HOS, the comparison between them might be of more sense. PC4 suppression decreased the invasion and migration abilities in 143B, and moreover, decreased the growth of 143B cells *in vitro* on plastic plates with an anchorage-dependent manner and in soft-agar with an anchorage-independent manner. Cell attachment ability was also declined with PC4 suppression. Downregulation of PC4 expression led to G1-phase arrest in 143B cells which may be responsible for the proliferation decline. PC4 presented anti-tumor growth effect and anti-lung metastasis effect in nude mice in the research which echoed the results *in vitro* experiments and clinical sample analysis.

RNA-seq analysis revealed us MMP9 and many tumor related changed genes might be the candidate targets for the PC4 tumor regulation in 143B. MMP9 is a hallmark gene of metastatic capability in many kinds of tumors. However, FN positively regulate MMP9 in many other tumors, and our RNA-Seq results suggest that PC4 has an effect on FN and MMP9 at the same time, so we checked the effect of exogenous FN on MMP9 in 143B. The results suggest that MMP9 in 143B is not sensitive to FN as we thought. So we speculate that PC4 regulate MMP9 directly. The following study confirmed our hypothesis, we demonstrated that PC4 shRNA downregulated MMP9 mainly through SP1 at the transcriptional level by forming the SP1 transcriptional complex, and this may contribute mechanistically to lung metastases in osteosarcoma.

Numerous metastasis-related genes were regulated by PC4, however, MMP9 did not seem to be the sole gene account for osteosarcoma lung metastasis. Other genes regulated by PC4 still have to be verified. And the research on the up-stream of PC4 in osteosarcoma might reveal us more inspiring information, which is already in our progress. In this report, we demonstrated PC4 is positively associated with the malignancy of osteosarcoma, especially in regards to lung metastasis ability. The purpose of this paper is to find a possible therapeutic target for osteosarcoma. Down regulation of PC4 in 143B depressed metastasis and proliferation, and showed potential therapeutic value. Our work highlights the need for further exploration on the specific functions of PC4 in osteosarcoma pathogenesis. The upstream mechanisms that result in PC4 overexpression in osteosarcoma remain unclear. Future studies may elucidate practical applications of PC4 as a biomarker for early diagnosis, as a therapeutic target for treatment, and as a predictor of patient prognosis. Although further work is required, our results have identified PC4 as a potential target for osteosarcoma treatment and related lung metastasis.

## MATERIALS AND METHODS

### Patients and specimens

Total 242 samples were analyzed. From January 2000 to July 2015, 82 samples were collected from patients at multiple centers with osteosarcoma who underwent surgical resection or biopsy. In addition, microarray sections from 116 osteosarcoma specimens and 44 normal adjacent tissues were bought from US Biomax, Inc. (Rockville, MD). Clinicopathological staging was assessed, according to the Enneking staging system [33]. Total 59 patients were successfully followed-up. All human tissue-related experiments were carried out with the approval of the ethics committee of the Third Military Medical University.

### Immunohistochemical staining

All the paraffinized fresh tissue sample sections (4μm) were deparaffinized, rehydrated. Antigens were retrieved with sodium citrate. Non-specific binding was blocked with PBS containing Triton X-100 and goat serum, and then reacted with human PC4 primary antibody (1:200; Cat. #sc-48778, Santa Cruz). Sections were incubated with secondary antibody (ZSGB-BIO, Beijing, China). Immunodetection was performed with DAB (ZSFB-BIO), and then counterstained with hematoxylin. The PC4 expression level was calculated by the intensity score and rate score of positive-staining cells as described previously [5].

### Cell lines and reagents

The 9901 was got from Dr. Wang Dong's lab in Daping Hospital and other cell lines were got from ATCC. Cell lines were cultured with the recommended medium (Gibco) and 10% fetal bovine serum.

### Lv-shRNA treatment for PC4 gene silencing and stable transfected clone selection

For stable lentivirus transfected clone selection, pLKD.CMV.RFP.U6.shRNA was used as the vector. Human Osteosarcoma cells were transfected with lv-shRNA against PC4 (NeuronBiotech, Shanghai, China; 5′-ACAGAGCAGCAGCAGCAGA-3′) with an appropriate amount of polybrene as previously described [31]. After 72h transfection, fluorescence microscopy was used to confirm the expression of tagged red fluorescence protein. Western-blot analysis and qPCR were conducted to confirm the efficiency of transfection. In order to assess pure, stable transfected clonogenic cells, limited dilution assay was used. Cells were diluted to a single cell per well into 96-well plates. Two weeks later, positive clones were picked with a fluorescence microscope for further experiments.

### Cell cycle assay

BD FACS (fluorescent-activated cell sorting) Calibur flow cytometer was used for cell cycle analysis according to the protocols of manufacturer. The cells to be tested were collected, centrifuged and fixed with ice-cold 70% ethanol, staining the DNA with 10g/ml propidium iodide for 30 min at 37°C in a solution containing 100mg/ml RNAase A, and followed by flow cytometry analysis.

### Soft agar colony formation

Agarose solution (Sigma) was compounded with DMEM medium and prepared into a 1.2% base agar layer before seeding cells into the top agarose layer. When reaching 80% confluence, cells were mixed at a density of 2500 cells/well with 0.6% top agarose solution added 10% FBS and 1% penicillin/streptomycin (Cat. #V900929, Sigma) in a 6-well plate. Fresh medium was added twice a week. Cells were cultured in soft agar medium for 3 weeks. Colony formation was assessed morphologically and quantified by the number of colonies formed per well.

### RNA-seq

Approximately 1×10^7^ 143B and 143B^PC4−^ cells were collected for RNA extraction according to the instructions of BGI. Concentration and quality of the RNA were checked. DNase I was used to degrade the possible DNA contamination of RNA samples. Then the oligo (dT) magnetic beads were used for the enrichment of mRNA. And the mRNA was fragmented, and then collected for hybridization and reverse transcription. The first strand of cDNA was synthesized by using random hexamer-primer. Then, the cDNA library was amplified according to the protocol, cleaned with magnetic beads. Sequencing adaptors were then linked to the fragments. The fragments were enriched by PCR by using Agilent 2100 Bioanaylzer and ABI StepOnePlus Real-Time PCR System. The library products were sequenced with Illumina HiSeqTM 2000. We got 12.5M reads with each sample. Gene expression was evaluated with the RPKM (Reads Per Kilobase per million reads) [34, 35]. P value was calculated accordingly. Corrections for false-positive were conducted with Benjamin's false discovery rate (FDR) [36]. We set p<0.01, FDR≤0.001 and the absolute value of log_2_Ratio (143B^PC4−^/143B) ≥1 as the threshold to identity the significance of different expressed genes. Functional classes were assigned by GO enrichment analysis, which was performed according to Gene Ontology, and Gene ontology functional classification of different expressed genes was performed on WEGO (Web Gene Ontology Annotation Plot)[37]. Pathway enrichment analysis of our mapping different expressed genes was performed with updated KEGG database (Kyoto Encyclopedia of Genes and Genomes).

### Reverse transcription-polymerase chain reaction (RT-PCR)

Cells RNA was extracted by RNAiso Plus according to the instructions (Cat. #9109, Takara). RNA was treated with DNAse from a PrimeScript® Kit (Cat. #RR047A, Takara) for 2 min at 42°C. cDNA was synthesized using SYBR® Premix Ex Taq™ (Takara CAT.# RR420A) with Applied Biosystem 7500 Fast quantitative real-time PCR system for 15 min at 37°C and 5 s at 85°C then 4°C. The qPCR reaction conditions were set as follows: 95°C for 1 min, followed by 45 cycles of 95°C for 10 s and 60°C for 34 s. GAPDH was set as control. Relative mRNA levels were calculated with 2^−ΔΔCT^ method.

### Western blotting analysis

Samples were lysed with RIPA buffer with 1 mmol/L PMSF (Beyotime). Protein concentration was evaluated with BCA kit (Beyotime). Proteins were loaded and separated by 10% (w/v) sodium dodecyl sulfate polyacrylamide gels (Bio-Rad) and transferred to 0.22 μm PVDF membrane (Millipore). Blocking buffer with 5% BSA in 1×TBST was used before immunoblotting. Protein blot bands on the membranes were detected with ECL chemifluorescence kit (Bio-Rad) on ImageQuant LAS 4000 (GE).

### Immunofluoresence staining

Cells were seeded onto confocal Petri dishes. After adherence, cells were washed with PBS, fixed with 4% paraformaldehyde. Then permeabilized with 0.25% Triton X-100 for 10 min, and blocked in 1% BSA. Cells were incubated with PC4 antibody (1:200) at 4°C overnight in a humidified chamber. Samples were incubated with fluorescence second antibody (1:1 000) for 1 h at room temperature in the dark. nucleuses were stained with DAPI. Images were captured with confocal microscopy.

### Cell growth assay

Cell proliferation was measured with the Cell Counting Kit-8 (Dojindo, Kumamoto, Japan). Cells were seeded into 96-well plates with a density of 2 000 cells each well and 100ul medium. Cells were allowed to attach overnight, and viability was tested at 0 h, 24 h, 48 h, and 72 h. Cellular proliferation was measured as fold-increase =(absorbance of tested group minus blank cells)/(absorbance of 0 h group minus blank group). Data were read by a microplate reader (Varioskan Flash Multimode Reader; Thermo Scientific). Tests were performed in quintuple.

### Scarification test

The cells were seeded into 6-well plates at a density of 3×10^5^ cells per well. After culture for 24h, wounds were generated in the cell monolayer with a 100ul pipette tip. The dead cells were removed by PBS washes. Cells were treated with serum-free medium for 24h, 48h, and 72h. Images were taken at the indicated time and then migration distance was measured.

### Transwell chamber assay

Cell inserts pre-coated with matrigel (6.0 μm pore size membrane; Corning) were used. Osteosarcoma cells deprived of serum (0.1%) for 12 hours, were seeded onto the upper chamber surface in serum-free medium at a density of 1×10^5^ cells per well, and 20% FBS was placed in the lower chamberand, and incubated at 37°C for 24 hours. At the end of incubation, nonmotile cells on the upper surface of the filter were wiped off and the cells on the underside chamber were fixed with methanol and stained with 0.1% Crystal Violet and counted using bright-field microscopy in 5 random fields in triplicate inserts.

### Adhesion assay

Cells pre-transfected with PC4 or vector lentivirus were serum starved overnight, seeded into 24-well culture plates (3×10^5^ cells/well in 0.5 ml of DMEM medium) and incubated at 37°C for 30 min, 60 min, 120 min, or 24 hours. After incubation, cells were slightly washed with PBS at the indicated time, and adherent cells were counted with CCK-8 kits.

### Tumorigenicity assays

Tumor model *in vivo* was established using 4-week-old male athymic nude mice from the Laboratory Animal Center of the Third Military Medical University (Chongqing, China) in a specific pathogen-free condition. Animal protocols were followed the Animal Care and Use Committee Guidelines of the Third Military Medical University. Mice were inoculated subcutaneously with 3×10^6^ respective osteosarcoma cells in 100 ul of PBS at one dorsal site. Tumor growth was grossly monitored and measured with sliding calipers every 2 days. Volume of tumors were calculated according to the formula: volume (mm^3^) = (width^2^ X length)/2. When mice were killed, tumors were dissected and weighed. Tumors and lungs were fixed in 4% paraformaldehyde, and sections were stained with hematoxylin and eosin (HE) for morphological examination. For the growth test, mice were sacrificed on day 27. For the pulmonary metastasis test, and to avoid the effect on proliferation, another 15 nude mice were used. Lungs were excised when tumor volumes reached 2 000 mm^3^

### Chromatin immunoprecipitation assay

Chromatin immunoprecipitation (ChIP) assays were performed according to manufacturer's instructions (Cat. #17-371, Millipore). Protein–DNA complexes were precipitated using a PC4 antibody (Santa Cruz) and PCR was carried out using following specific primers: MMP9, forward: 5-CTACTGTCCCCTTTACTGCCCTGAA-3; reverse: 5- TCCCAGGTCAGATATCCTCCCCAA-3. Genomic DNA and IgG were used as controls.

### Luciferase reporter assay

Luciferase assays were performed according to the manufacturer's instructions (Promega CAT.# E2510). The human MMP9 promoter Luc reporter which construced by human MMP9 promoter (−700,−100) and Luc reporter construct (PGL 4.20) (Addgene, Cambridge, MA) was developed by Dr Zhuang’ lab (SNB, NINDS, NIH). The Sp1 siRNA (Cat. # sc-90024, Santa Cruz) and PC4 siRNA (Cat.# sc-38583, Santa Cruz). Transfections were performed using Lipofectamine 2000 (Cat. #12566014, Invitrogen). Normalizing the DNA amount in each well with empty vector DNA, and the luciferase activity was normalized to light units per microgram protein. Experiments were performed in triplicate.

### Co-immunoprecipitation

Immunoprecipitation was performed as manufacturer's instructions (Cat. #26149, Thermo Scientific). Briefly, cells were lysed in RIPA buffer with Protease Inhibitor Cocktail and 0.5% SDS. Total cell lysate was precipitated with the DynaBeads Protein G Immunoprecipitation Kit (Cat. #10007D, Novex) and antibodies against PC4 (Cat. #sc-48778, Santa Cruz), antibodies against SP1 (Cat.# 5931, CST). Precipitated protein was eluted and detected by Western blot.

### Statistical analysis

Data were given as mean ± standard deviation, and analyzed by SPSS 16.0 or GraphPad Prism 5.0 (GraphPad software). Chi-square test and Fisher exact test were used for comparing the expression of PC4 in each group. Kaplan-Meier survival curves were compared by the Gehan-Breslow-Wilcoxon Test. p < 0.05 was considered to be of statistical significant.
